# Demonstrating the Connection between the Nonvalence Correlation-Bound Anions of Polyaromatic Hydrocarbons and the Image Potential States of Graphene Using a One-Electron Model Hamiltonian

**DOI:** 10.1021/acs.jpclett.4c01308

**Published:** 2024-06-10

**Authors:** Devin M. Mulvey, Kenneth D. Jordan

**Affiliations:** Department of Chemistry, University of Pittsburgh, 219 Parkman Avenue, Pittsburgh, Pennsylvania 15260, United States

## Abstract

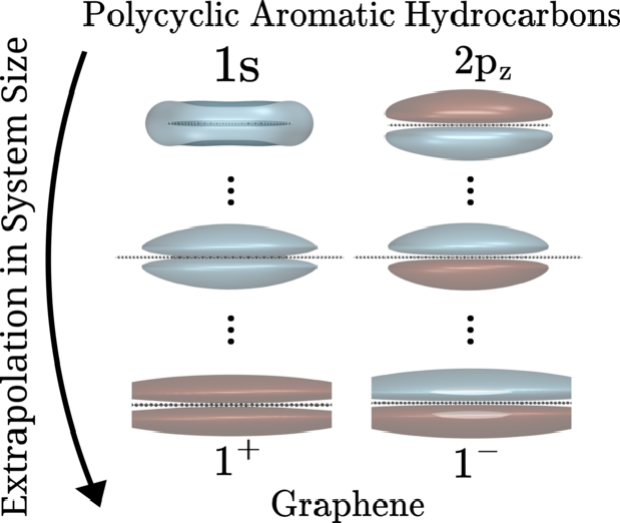

The ground and excited state nonvalence correlation-bound (NVCB) anion states of the  hexagonal polycylic aromatic hydrocarbons and of hexagonal  graphene nanoflakes are characterized using a one-electron model Hamiltonian which incorporates atomic electrostatic moments up to the quadrupole, coupled inducible charges and dipoles, and atom-centered Gaussians to describe the short-range repulsive interactions. Extrapolation of the calculated electron binding energies of the lowest energy symmetric and antisymmetric (with respect to the molecular plane) NVCB anions of both the polycylic aromatic hydrocarbons and the carbon nanoflakes to the *n* → ∞ limit yields binding energies that are in good agreement with those of the most stable symmetric and antisymmetric image potential states of freestanding graphene as determined from two-photon photoemission spectroscopy (2PPE) experiments.

Nonvalence correlation-bound (NVCB) anions constitute a class of stable anions that are markedly different than their valence counterparts.^1−27^ While short-range interactions stabilize valence anions, long-range, “dispersion-like” correlation effects involving configurations in which both the excess electron and a valence electron of the neutral molecule are simultaneously excited, stabilize NVCB anions. The Hartree–Fock (HF)^28−30^ method and post-HF methods that depend on HF providing a good starting wave function fail to bind NVCB anions. In a many electron treatment it is essential to include electronic configurations that allow the orbital occupied by the excess electron to “relax” in response to dispersion-type correlation.^11,12^ Theoretical studies have predicted^3−19^ and experimental studies have confirmed the existence of NVCB anions for a variety of molecules and molecular clusters.^20−27^

NVCB anions have also been characterized by use of one-electron model Hamiltonians that incorporate polarization of the molecule or cluster by the excess electron.^5,6,9,10^ In a previous publication, we characterized the lowest energy NVCB anion of  (*n* = 3–7) hexagonal polycylic aromatic hydrocarbons PAHs using a one-electron model Hamiltonian.^8^ In the present study we extend this earlier work to larger hexagonal (PAHs) and to graphene nanoflakes (GNs), with the goal of examining the evolution of the NVCB anions of these species into the image potential states of graphene. In addition, we also report results on the excited NVCB anion states of these systems.

A point charge (*q*) at a distance *z* above the surface (assumed to lie in the *xy* plane) of a conductor experiences an image potential *V*(*z*) = −*q*^2^/4*z* (in atomic units), which derives from the collective polarization response of the conductor’s electrons to the presence of the external charge.^31^ The image potential supports a series of bound anionic states, referred to as image potential states (IPSs), in which the excess electron is confined in the *z* direction, but free in the *x* and *y* directions.^32^ It has been suggested that the NVCB anions and IPSs are related since in a many-body treatment the excess electron is bound by dispersion-like correlation effects for both species.^6,7,12,33^

The *V*(*z*) = −*q*^2^/4*z* potential is only valid in the positive half space above the conductor (*z* > 0). While this is appropriate for graphene on a supporting substrate, in the case of free-standing graphene the polarization potential is attractive in both half spaces, making *V*(*z*) = −*q*^2^/|4*z*| the appropriate potential. As a result, an excess electron binds to both faces of graphene’s atomic plane producing two series of IPSs, one with wave functions symmetric and the other with wave functions antisymmetric with respect to reflection in the plane of the carbon atoms.^34−38^ For the unmodified –*q*^2^/4|*z*| potential, the energy of the lowest energy symmetric state diverges.^39,40^ However, this is not an issue for realistic systems, for which the potential does not diverge as *z* → 0.

The IPSs of graphene have been calculated using periodic boundary conditions with the short-range exchange-correlation potential of a local DFT (LDA) combined with the long-range image potential.^34,35^ Alternatively, de Andres et al. opted to represent graphene as a polarizable dielectric described via Thomas-Fermi theory or the random phase approximation (RPA) in a one-dimensional one-electron model, which exhibits the correct –*q*^2^/4|*z*| asymptotic image tail and avoids the singularity at short-range.^41^ However, these approaches are not applicable to the finite systems of interest here for which the asymptotic potential deviates from –*q*^2^/4|*z*|, with the extent of the deviation depending on the values of *x* and *y*.

In the present study we apply a one-electron Hamiltonian to characterize the NVCB anions of hexagonal PAHs and GNs as large as C_5400_H_180_ and C_5400_, respectively. The EBEs obtained from these calculations are used to extrapolate to the *n* = ∞ limit, allowing us to assess convergence to the graphene limit.

The model Hamiltonian used in this study is of the form,^6,8−10,42^
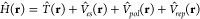
1where  is the kinetic energy operator, , , and  represent, respectively, the electrostatic, polarization, and repulsion potentials that the excess electron (positioned at **r**) experiences in the presence of the molecule.

To represent the static charge densities of the PAHs up to C_216_H_36_ in size we use atomic charges, dipoles, and quadrupole moments derived from Gaussian distributed multipole analysis (GDMA)^43−45^ of PBE0^46−49^ densities calculating using the cc-pVDZ basis set.^50^ For the larger PAHs we use the multipole moments as described in the SI. For the GNs, electrostatics were ignored. This is justified by the fact that for an infinite array of quadrupoles associated with the C atoms of an infinite graphene sheet the electrostatic interaction with a point charge is zero.^51,52^ For real graphene samples edge termination necessarily introduces edge dipoles. However, since the primary goal of the calculations on the GNs is to provide results to enable extrapolation to infinite system size, we considered it appropriate to ignore edge effects in these calculations.

To model the polarization response of the PAHs to the excess electron we use an inducible charge and dipole model adapted from the work of Mayer and Åstrand.^53,54^ Two slightly different models designated modified-Mayer-Åstrand (MMÅ1) and modified-Mayer-Åstrand two (MMÅ2) are employed. The MMÅ1 model has been shown to accurately describe the dipole polarizabilities and polarization potentials of small PAHs.^8^ The MMÅ2 model, which includes an additional term damping the short-range interaction between induced atomic charges, shows better convergence to the classical image potential for large *z* values as *n* → ∞.^42^ The orthogonalization of the orbital occupied by the excess electron to the occupied orbitals of the neutral molecule, charge penetration, and exchange effects are folded into the repulsive potential, which is modeled via atom-centered Gaussians as described in ref.^8^

The energy of the excess electron is evaluated on a real space grid of points, **r**, using a sine-type discrete variable representation (DVR) basis.^6,8−10^ Well converged energies are obtained with grid-point spacing of 1 Bohr, although due to program limitations, for the largest systems considered, we adopted a grid spacing of 2 Bohr, which introduces small (few percent) errors in the calculated EBEs. Care was taken to ensure that the box sizes employed were large enough to achieve convergence.

We consider first the NVCB states of the PAHs  (*n* = 3–7). Although these “small” systems will not be used in extrapolating the EBEs to the infinite system limit, we include them here as the information on their NVCB anion states should be of considerable interest to experimentalists investigating these species. Calculations were performed for these PAHs using both the MMÅ1 and MMÅ2 models.

The smallest hexagonal PAH that has been predicted to have a bound NVCB anion is circumcoronene (C_54_H_18_).^7,8^ For this species, only a single bound NVCB anion state is found. Due to the absence of nodes, we label this state as 1*s*-like. Note that in a many-electron treatment this orbital would have nodes to ensure orthogonality to the valence and core orbitals of the same symmetry. The MMÅ1 model was parametrized to reproduce the EOM-MP2 value of the EBE of 12.3 meV^7^ for the NVCB anion circumcoronene. The MMÅ2 model gives an EBE of 17.1 meV for circumcoronene. The larger PAHs displayed in Figure 1 also have bound excited NVCB anion states with one or more nodal surfaces perpendicular to the *xy* plane. In particular, C_96_H_24_ has *p*-like NVCB anion states, C_150_H_30_ supports *p*- and *d*-like NVCB anion states, and C_216_H_36_ and C_294_H_42_ support *p*-, *d*-, and *f*-like NVCB anion states. For the high-lying eigenvalues, corresponding to *f*-like states, there is a small splitting of the energies of states that should be degenerate. This also occurs for some of the larger molecules considered below, for which there are some cases of broken symmetry. These issues appear to be numerical in nature, reflecting problems in the diagonalization as a result of multiple eigenvalues falling close in energy. However, in extrapolating the results to the graphene limit, we will be using eigenvalues for which these issues do not arise.

**Figure 1:**
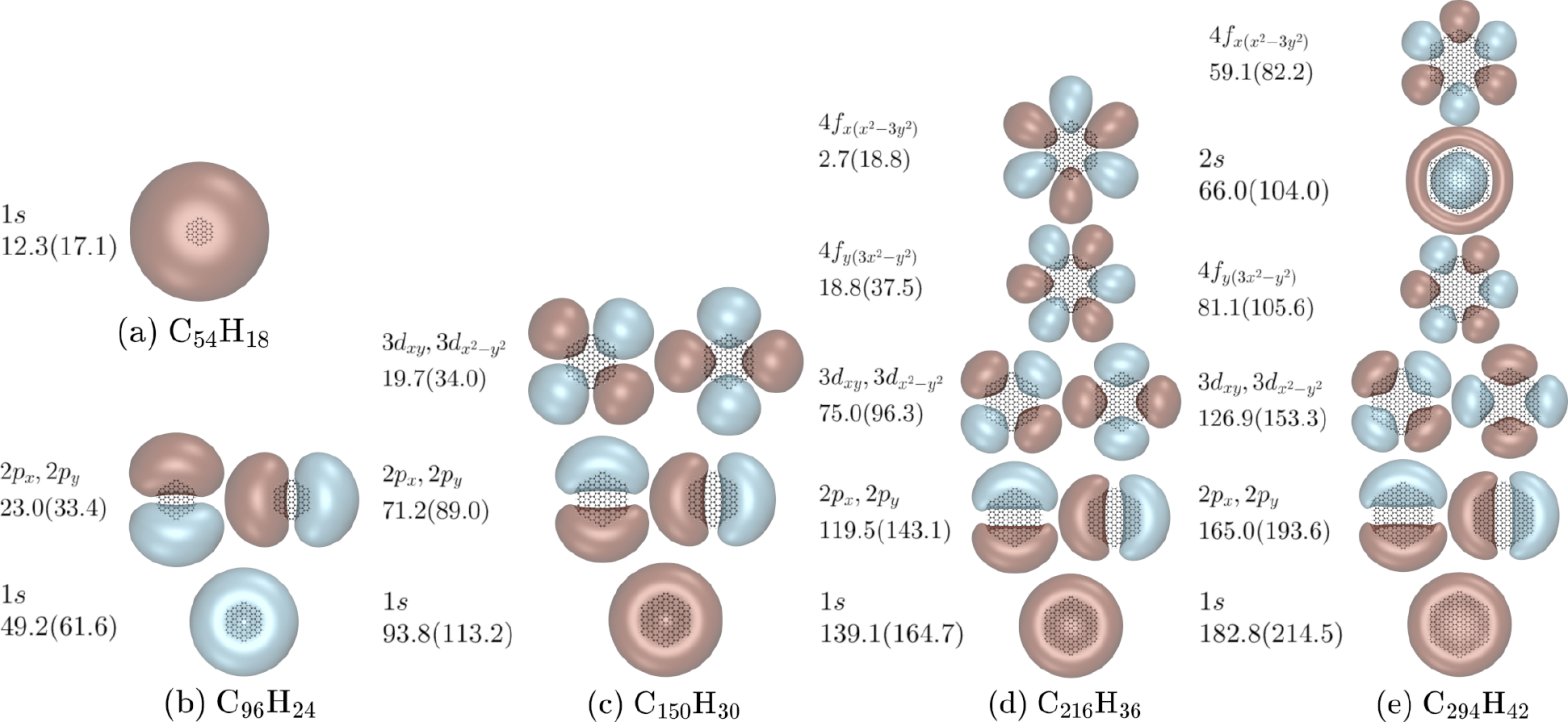
EBEs in meV and single particle orbitals for NVCB anion states of the PAHs: C_54_H_18_, C_96_H_24_, C_150_H_30_, C_216_H_36_, and C_294_H_42_. Binding energies are reported for both the MMÅ1 and MMÅ2 polarization models, with the latter values being given in parentheses. The orbitals are from the calculations using the MMÅ1 polarization model. The orbitals are plotted at 90% charge enclosure of the excess electron’s density and are labeled based on the hydrogenic orbitals that they resemble. These plots, and those presented in subsequent figures, were made using VMD.^55^

Significantly, our model potential calculations predict that none of the PAHs up to C_294_H_42_ support a NVCB anion state with a node in the plane of carbon atoms. This is largely a consequence of the electrostatic term in the potential which is attractive near the H atoms. This favors states without a node in the *xy* plane as demonstrated by calculations on C_150_H_30_, C_216_H_36_, and C_294_H_42_ with the electrostatics omitted from the potential. In the absence of electrostatic interactions, the calculations do give a bound 2*p*_*z*_-like NVCB anion state for these species.

As noted above, the MMÅ1 model gives a binding energy for the 1*s* NVCB anion of C_54_H_18_ close to the EOM-MP2 result of ref (7), while the MMÅ2 calculations yield a larger EBE. While inclusion of electron correlation effects not recovered in EOM-MP2 could lead to an even larger EBE for the 1*s*-like NVCB anion of C_54_H_18_,^11,12^ we expect that for this species, the MMÅ1 value of the EBE is closer to the true value than that obtained using MMÅ2. This is rationalized on the basis that MMÅ2 slightly overestimates the polarizabilities of the small PAHs. More importantly, as the size of the PAHs approach the nanoscale the MMÅ2 model shows better convergence to the image potential and concomitant induced charge density.^42^ With this in mind, the results reported from this point forward make use of the MMÅ2 induced charge polarization model.

We now consider the NVCB anions of the C_600_, C_2400_, and C_5400_ GNs and the C_600_H_60_, C_2400_H_120_, and C_5400_H_180_ PAHs. Figures 2, 3, and 4 report the orbitals and EBEs of the low-energy bound NVCB anion states of these species as described by the Hamiltonian with the MMÅ2 polarization model.

**Figure 2:**
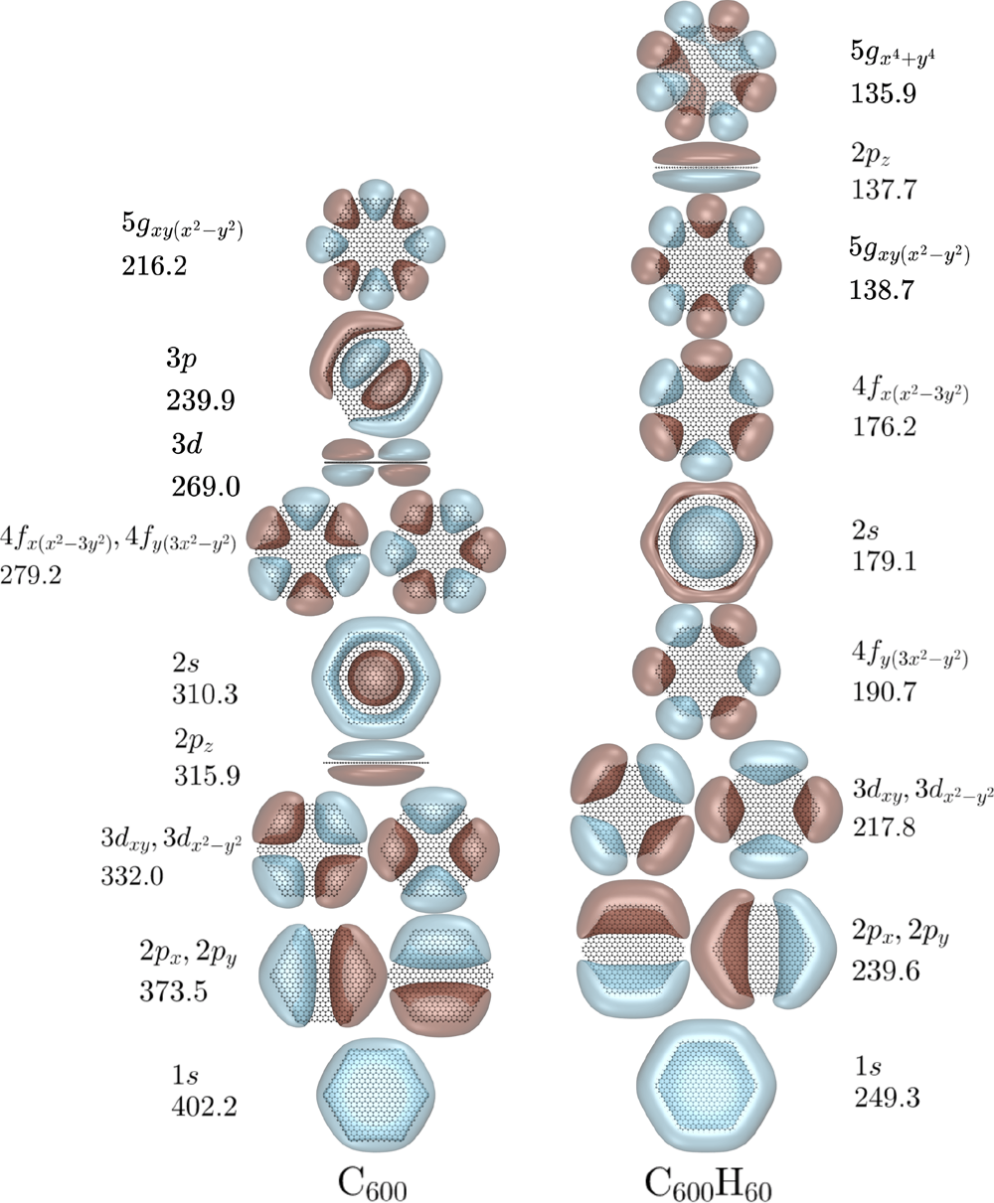
EBEs in meV and single particle orbitals for the NVCB anion states predicted for C_600_ and C_600_H_60_. Orbitals are plotted at 90% charge enclosure of the excess electron’s density. The orbital labels are based on the hydrogenic orbitals that they resemble.

**Figure 3:**
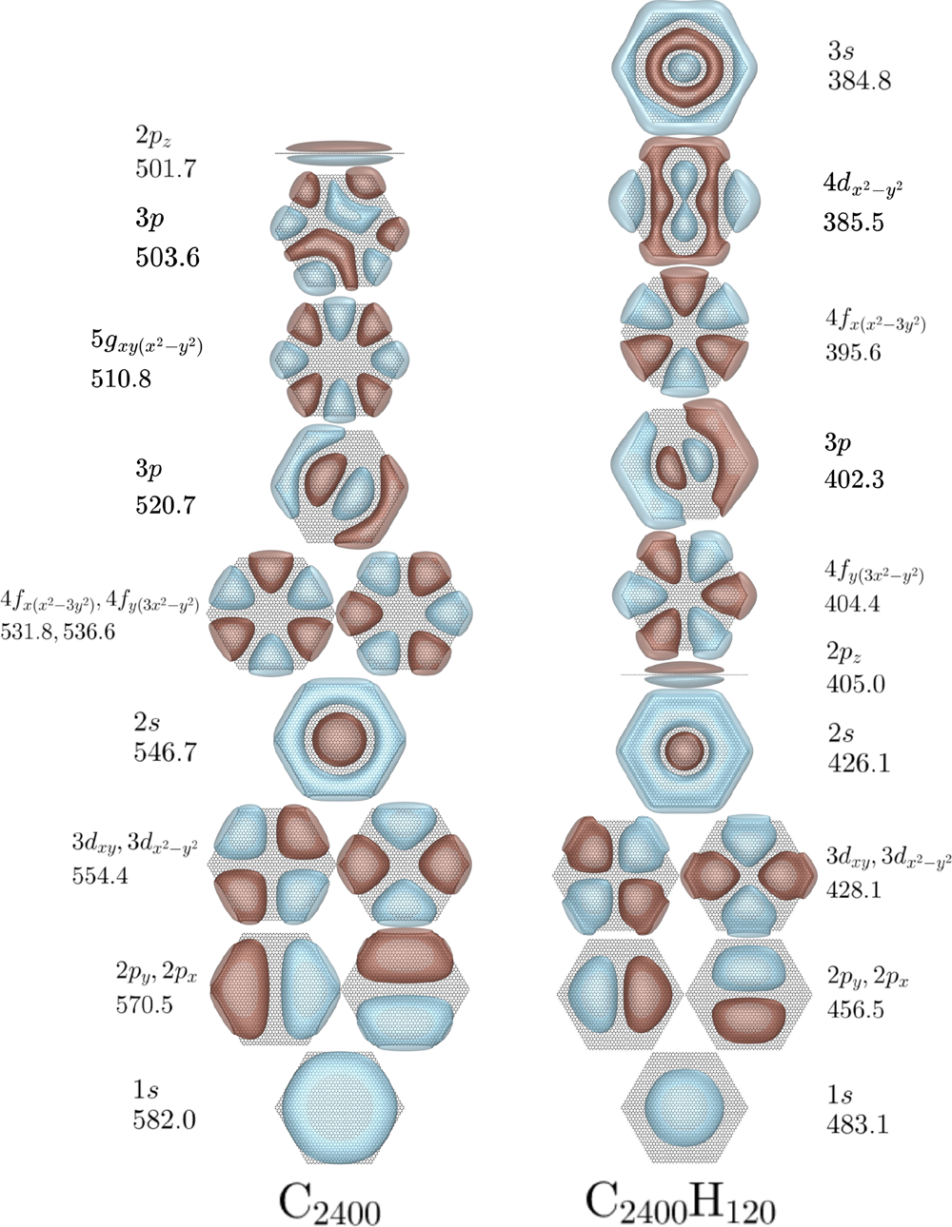
EBEs in meV and orbitals for the NVCB anion states predicted for C_2400_ and C_2400_H_120_. See Figure 2 for details regarding the symmetry labels.

**Figure 4:**
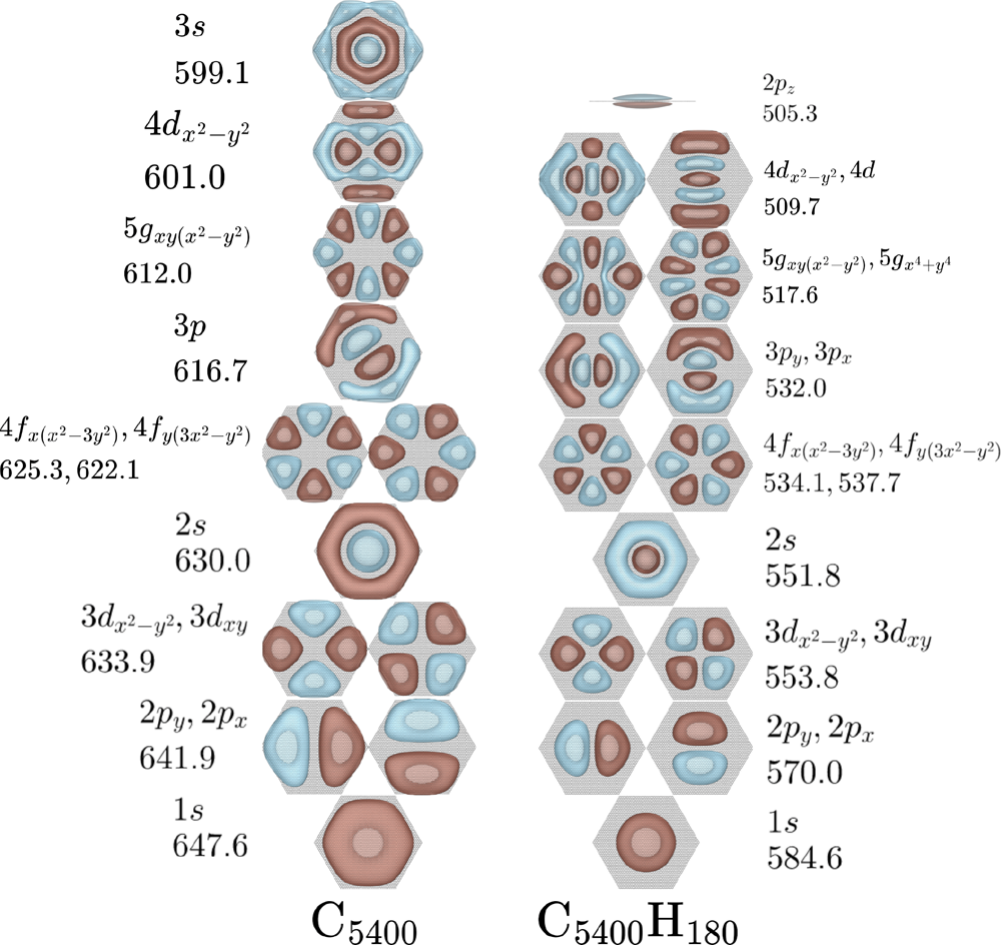
EBEs in meV and single particle orbitals for the NVCB anion states predicted for C_5400_ and C_5400_H_180_. See Figure 2 for details regarding the symmetry labels.

From Figures 2, 3, and 4 one sees that the various NVCB anion states of both the GN systems and the PAHs are stabilized with increasing molecular size. This is a consequence of the polarization potential becoming more attractive and the energy penalty for the confinement of the excess electron in the *x* and *y* directions becoming less important with increasing molecular size. Moreover, the EBEs obtained with and without inclusion of electrostatics approach one another with increasing molecular size. Consistent with this is the observation that the amount of electron density near the CH groups decreases with increasing size of the PAH. For some of the high energy NVCB anion states, e.g., the 3*p*_*x*_ and 3*p*_*y*_ pair for C_600_, our calculations find only one member of the degenerate pair.

We now consider the effect of confinement on the energies of the various NVCB anion states with no node in the carbon atom plane of the GN and PAH species. Since the systems considered have 6-fold symmetry, we expect that the effect of confinement would be well described by the electron in a circular box problem with a potential constant inside the box and infinite outside. The eigenvalues of the circular box problem go as  (in atomic units), where *R* is the radius and *z*_*nm*_ are given by the nodes of the Bessel functions. Subscripts *n* and *m* are the two relevant quantum numbers related to the number of radial and angular nodes, respectively.^56^

In Table 1, we report the energies of the various excited NVCB anion states relative to the lowest energy NVCB anion state of C_600_H_60_ and C_600_, as well as the energy differences between the excited states and the ground state for the electron in the circular box problem with *R* = 61 Bohr. This value of *R*, while approximately 30% larger than the distance from the center of the ring to the midpoint of the C atoms on the perimeter closest to the *x*-axis, gives the best agreement with splittings from the model potential calculations on C_600_. The need to employ an *R* value considerably larger than the radius of the ring is consistent with the fact that for C_600_, the distribution of the excess electron extends (in the *xy* plane) to distances appreciably further than the carbon atom plane’s edge.

**Table 1:** Differences of energies between the excited and lowest energy NVCB anion states of C_600_H_60_ and C_600_ and the energy differences for the electron in the circular box (CB) problem. Energies are in meV

Excited state	CB	C_600_	C_600_H_60_
2*p*	34	29	10
3*d*	75	70	32
2*s*	90	92	70
4*f*	128	123	59
3*p*	159	162	-
5*g*	190	186	111

One sees from the results in Table 1 that the energy spacings of the NVCB anion states of C_600_ obtained from using the MMÅ2 model are nearly identical to those from the electron in the circular box problem. This is also true for C_2400_ and C_5400_ with appropriate choices of the radii for the circular box. Of particular note is the finding that the energy ordering of the levels, 1*s* < 2*p* < 3*d* < 2*s* < 4*f* < 3*p*, ..., for the circular well problem is the same as predicted for the C_600_ and C_2400_ GNs as described by our one-electron model Hamiltonian in the absence of electrostatics. With the inclusion of electrostatics, the agreement between the relative energies of the various NVCB anion states from the model potential and the particle in the circular box problem is less quantitative.

To better understand the impact of hydrogen termination on the NVCB states on large PAHs, we plot in Figure 5 slices of our model potential with (PAHs) and without (GNs) electrostatics. One-dimensional slices of the potentials are taken along the *y*-axis at *z* = 3, 6, and 9 Bohr above the carbon atom plane of GNs C_600_ and C_2400_ and PAHs C_600_H_60_ and C_2400_H_120_.

**Figure 5:**
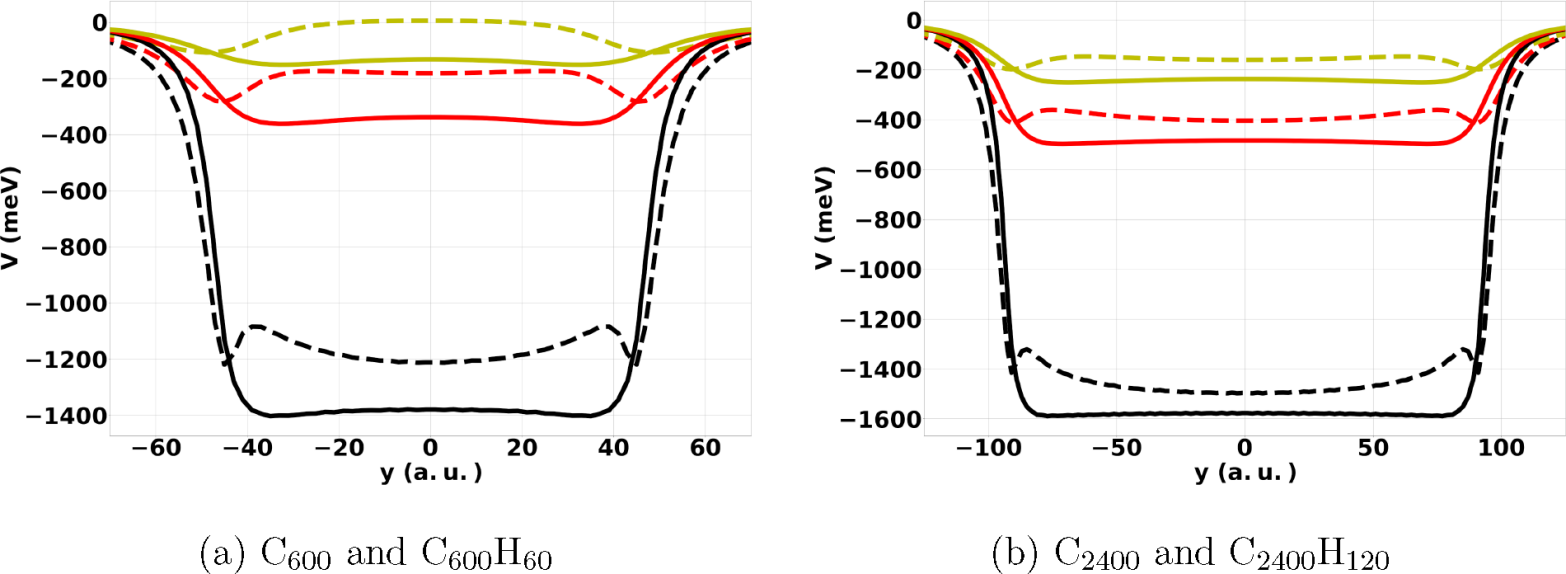
One-dimensional slices along the *y*-axis of the potential for a negative point charge interacting with GNs: C_600_ and C_2400_ as well as PAHs: C_600_H_60_ and C_2400_H_120_. Solids lines represent the potential associated with GNs and dashed lines the potential associated with PAHs. The distance of the slice above the plane of atoms (*z*) is fixed at 3 (black lines), 6 (red lines), and 9 (yellow lines) Bohr.

Figure 5 illustrates how the inclusion of electrostatics causes a shift in the charge density of the excess electron toward the hydrogen terminated edges of small PAHs. In the absence of electrostatics, the potentials for interaction of the point charge at a fixed height above the surface with the molecules are approximately constant out to a radius slightly larger than the molecular radius. The electrostatic potential is repulsive above and below the C atoms, but is attractive near the H atoms. Comparison of the potentials for C_600_H_60_ and C_2400_H_120_ reveals that with increasing size of the PAH the destabilizing effect of electrostatics above and below the C atoms becomes less important relative to the stabilization provided by polarization.

We now examine the convergence of the binding energies of the 1*s* and 2*p_z_* NVCB anion states of the  and  species as *n* → ∞. Our focus is on the 1*s* and 2*p_z_* NVCB anion states as they are the finite system analogs of the most stable symmetric (1^+^) and antisymmetric (1^–^) IPSs of free-standing graphene. To accomplish this, we extrapolate the EBEs of the *n* = 10, 12, 15, 20, 25, and 30  PAHs and  GNs. We consider two different fits, one using data for all six of these *n* values and using the expression EBE = *A* + *B*/*n* + *C*/*n*^2^, and the other fitting only the first two terms of this expression (*A* and *B*) to the EBEs of the *n* = 20, 25, and 30 species. Note that our calculations did not yield a 2*p*_*z*_-like NVCB state for C_5400_ within the first 25 eigenstates. As a result, both extrapolation procedures for the 2*p*_*z*_-like root of the GNs lack this data point.

The extrapolated intercepts of the polynomial fits in Figure 6, give our model estimates of the binding energies of the 1^+^ and 1^–^ IPSs. As seen from the figure, the EBEs of the  species (i.e., in the absence of electrostatics) are nearly linear in 1/*n*, with the two fits providing nearly the same estimates of the EBEs of the 1^+^ and 1^–^ IPSs. In contrast, the EBEs for the PAHs show more curvature when plotted against 1/*n*. As a result, the two fitting procedures to the EBEs of PAHs give somewhat different estimates of the IPSs of graphene. This is a consequence of the electrostatic contributions to the potential. In going forward, we focus on the EBE = *A* + *B*/*n* fits to the EBEs for the three largest systems considered, for which electrostatics is less important. For these larger systems the linear fits of the EBEs of the  PAHs and  GNs give nearly the same estimates of the 1^+^ and 1^–^ IPSs.

**Figure 6:**
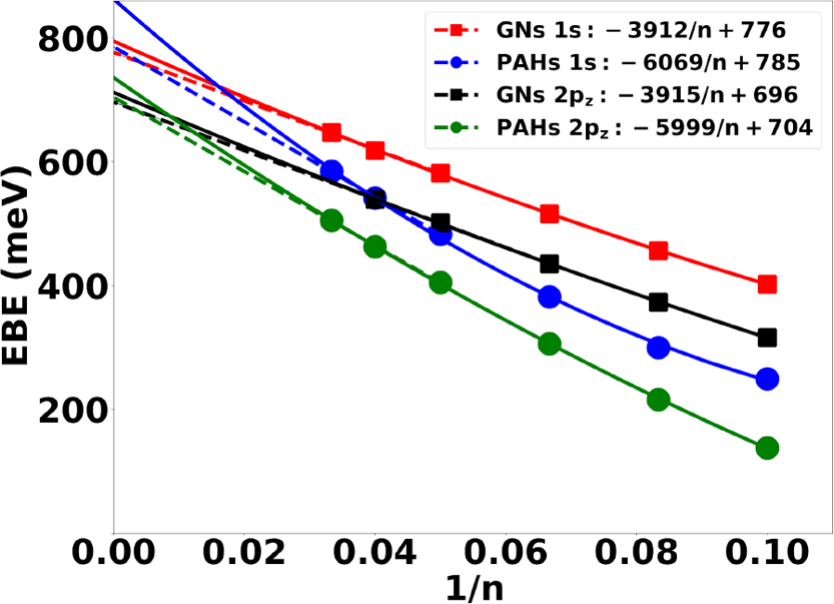
Extrapolation of one-electron model values of the EBEs for the 1*s* and 2*p*_*z*_ NVCB anions of the  PAHs and  GNs with respect to 1/*n*. The fits to all six data points are represented by solid curves, and those fitting to only the *n* = 20, 25, and 30 species are represented by dashed curves. Filled circles and squares represent the EBEs of  PAHs and  GNs, respectively.

Table 2 compares the IPS binding energy estimates obtained from the extrapolation of the calculated EBEs for the *n* = 20, 25, and 30 PAHs to calculated^34,35^ and experimental^37,38^ literature EBEs for the 1^+^ and 1^–^ IPSs of graphene. As can seen from Figure 6, nearly identical results are obtained from the fits of the EBEs for the  species, so these are not reported separately in Table 2.

**Table 2:** Electron Binding Energies in eV of the 1^+^ and 1^–^ IPSs of Graphene As Predicted by Extrapolation of the EBEs of NVCB Anion States of PAHs^a^

	One-electron model	LDA+Image Potential^b^						2PPE Exp.	
IPS		(34)			ref (35)				
		1.6	2.1	2.6	1.6	2.1	2.6	ref (37)	ref (38)
1^+^	0.78	1.47	1.33	1.29	1.43	1.30	1.27	1.05	1.04 ± 0.1
1^–^	0.70	0.72	0.61	0.57	0.64	0.52	0.49	0.78	0.77 ± 0.1

a Also included are the EBEs calculated using DFT^34,35^ and experimental results from two-photon photoemission spectroscopy (2PPE).^37,38^.

b Refs (34) and (35) defined the image plane to be *z*_*im*_ = 0 and *z*_*im*_ = 1.048 Å, respectively. Both studies considered values of *z*_0_ = 1.6, 2.1, and 2.6 Å for matching the image potential with the short-range potential of LDA.

As seen from the results reported in Table 2, the EBEs from our extrapolation procedure are in good agreement with the experimental values for the binding energies of the IPSs of free-standing graphene. For the 1^+^ state, extrapolation of the EBEs of the  PAHs gives an EBE of 781 meV, as compared to the experimental value of 1040 ± 100 meV. For the 1^–^ state, the extrapolation procedure gives an EBE of 700 meV, which is in excellent agreement with the two reported experimental values (780 and 770 ± 100 meV). The underestimation of the EBEs of the graphene IPSs obtained by the extrapolation of the model potential results for finite PAHs could be a consequence of a too repulsive short-range potential in the model. We note that the various DFT calculations give a much stronger binding of the 1^+^ state of graphene that predicted from our extrapolation procedure or found experimentally. This may reflect a deficiency in the DFT calculations in describing the short-range electron-graphene interactions, which are more important for the more tightly bound 1^+^ state.^41^

In this article, we used a one-electron model Hamiltonian to characterize the nonvalence anion states of a series of hexagonal  polycylic aromatic hydrocarbons and  graphene nanoflakes. Extrapolation of the EBEs from our calculations to the infinite size limit gives electron binding energies in good agreement with the energies of the 1^+^ and 1^–^ IPSs of graphene as determined from two-photon photoemission spectroscopy experiments. In addition, we show that the symmetry ordering and relative energies of the various excited NVCB anion states of the finite systems can be understood as a confinement effect which is well modeled, especially in the absence of electrostatics, by the electron in a circular box problem. The PAHs considered here have bound valence anion states. As a result, it should be possible to access a subset of the NVCB anion states by electronic absorption from the valence ground state anion.

## Supporting Information Available

Procedure used to produce the quadrupole+edge dipole atom centered multipole electrostatic model of large PAHs (PDF)
