# Supplementary material for: Demonstrating the Connection between the Nonvalence Correlation-Bound Anions of Polyaromatic Hydrocarbons and the Image Potential States of Graphene Using a One-Electron Model Hamiltonian
Source: J Phys Chem Lett. 2024 Jun 10;15(24):6299–305. doi: 10.1021/acs.jpclett.4c01308 (PMC11194819; doi:10.1021/acs.jpclett.4c01308)
Supplement: Supplementary file 1 — jz4c01308_si_001.pdf [file jz4c01308_si_001.pdf]

Supporting Information for "Demonstrating the Connection between the Nonvalence Correlation-Bound Anions of Polyaromatic Hydrocarbons and the Image Potential States of Graphene Using a One-Electron Model Hamiltonian"

Devin M. Mulvey* and Kenneth D. Jordan*

Department of Chemistry, University of Pittsburgh, Pittsburgh, Pennsylvania, 15260, United States of America

E-mail: dmm219@pitt.edu; jordan@pitt.edu

In this document we provide a brief description of the method by which we produced the quadrupole+edge dipole atom centered multipole electrostatic model used to calculate the NVCB anions of $n = 10,\ 12,\ 15,\ 20,\ 25,$ and 30 $C_{6n^2}H_{6n}$ PAHs in the main manuscript.

The electrostatic model is derived from basis space iterated Stockholder atoms (BS-ISA)[1,2] multipoles calculated using CamCASP v.7.2.2.[1,2] The BS-ISA multipoles were partitioned from asymptotically corrected[3] PBE0[4,5] electron density calculated via Psi4 v.1.4a2.dev213.[6–8] The PBE0 calculations were density fitted, using the orbital basis aug-cc-pVTZ[9–11] and the SCF-auxiliary basis cc-pVTZ (with diffuse functions added) for Coulomb-exchange fitting (-JK).[12]

The BS-ISA algorithm also uses density fitting, employing auxiliary bases for both the molecular density and atomic densities. We developed our own auxiliary basis for fitting the molecular density, which is based upon and further augments aug-cc-pVTZ-RI,[13] while aug-cc-pVQZ-RI was used for the atomic densities. The s-block of the molecular and atomic density fitting bases were replaced by the ISA set2 s-functions developed by the authors of BS-ISA.[1]

Further details regarding the multipole calculations are outside the scope of this work and will be made available in a separate publication which is currently in preparation.[14] What we will now describe is how the BS-ISA multipoles were used to generate an electrostatic model which uses both atomic out-of-plane quadrupoles Q_{20}^{C} and edge dipoles (Q_{1m}^{CH}). The Q_{20}^{C} component comes directly from the innermost carbon atoms of the largest system we could calculate BS-ISA moments for ($C_{96}H_{24}$). The edge dipoles were produced in a different manner, which went as:

1. Construct Q_{1m}^{CH} for C_{24}, C_{54}, and C_{96} such that the combination of Q_{20}^{C} on every carbon atom and Q_{1m}^{CH} on only the edge carbon atoms of the nanoflakes reproduce the molecular out-of-plane quadrupole of the PAHs $C_{24}H_{12}$, $C_{54}H_{18}$, and $C_{96}H_{24}$ when the $\ell = 2$ expansion (atomic charges, dipoles, and quadrupoles) of BS-ISA is used.

2. Plot the edge dipole magnitude of C_{24}, C_{54}, and C_{96} as a function of 1/r where r is the average distance of an edge carbon from the center of mass of the PAHs (determined using a nearest-neighbors algorithm).

3. Fit the function $Q_{1m}^{\mathrm{CH}}(1/r) = a*(1/r)+b$ to the data using the Python library NumPy's[15] `polyfit` function and extrapolate to obtain edge dipole magnitudes for the hexagonal carbon nanoflakes in the series C_{6n^2} $n = 2, 3, \ldots, 50$. Figure S1 plots the results of the extrapolation procedure and the constructed edge dipoles.

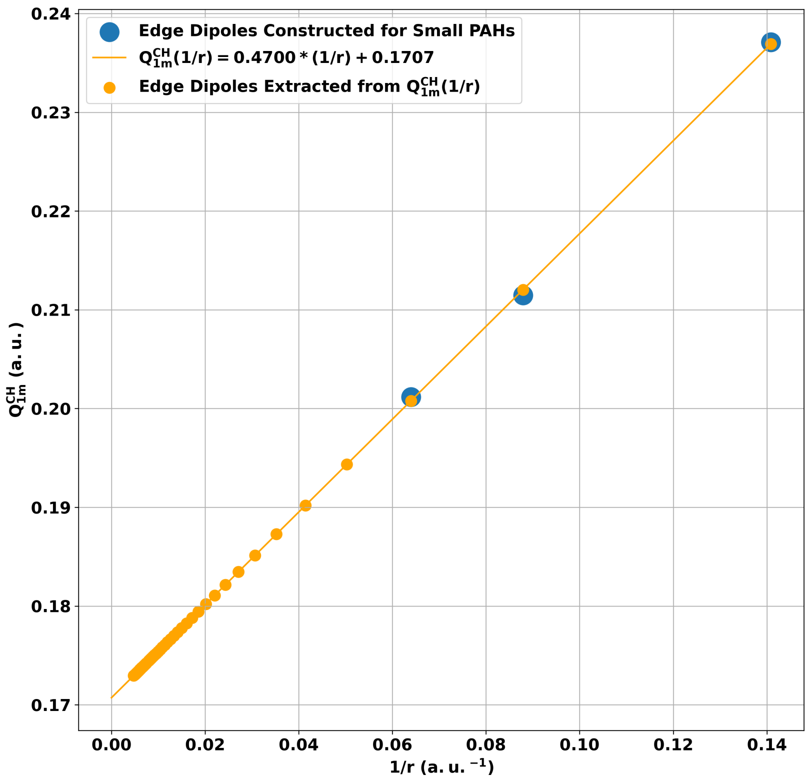

Figure S1: Plot of model edge dipole magnitudes (Q^{CH}_{1m}) as a function of $1/r$ where r is the average radius of a nanoflake. The blue points are the edge dipoles we constructed to reproduce the molecular out-of-plane quadrupole component of PAHs $C_{24}H_{12}$, $C_{54}H_{18}$, and $C_{96}H_{24}$. The function $Q^{\mathrm{CH}}_{1m}(1/r)$ was fit to the blue points and the orange points are the model edge dipoles extracted from the function.

4. Generate a moments file for the hexagonal carbon nanoflakes C_{6n^2} n = $2, 3, \ldots, 50$ where Q^{C}_{20} is placed on every atom and the edge dipoles determined in the previous step are applied to the edge carbon atoms with the correct orientation. In this context, "correct orientation," refers to the fact that the edge bond dipoles from BS-ISA point along the C-H bond towards the position of where a hydrogen atom would be in a PAH of the same size.

References

(1) Misquitta, A. J.; Stone, A. J.; Fazeli, F. Distributed Multipoles From a Robust Basis-Space Implementation of the Iterated Stockholder Atoms Procedure. *J. Chem. Theory Comput.* **2014**, *10*, 5405–5418.

(2) Misquitta, A. J.; Stone, A. J. ISA-Pol: Distributed Polarizabilities and Dispersion Models From a Basis-Space Implementation of the Iterated Stockholder Atoms Procedure. *Theor. Chem. Acc.* **2018**, *137*, 1–20.

(3) Grüning, M.; Gritsenko, O. V.; Van Gisbergen, S. J. A.; Baerends, E. J. Shape Corrections to Exchange-Correlation Potentials by Gradient-Regulated Seamless Connection of Model Potentials for Inner and Outer Region. *J. Chem. Phys.* **2001**, *114*, 652–660.

(4) Adamo, C.; Barone, V. Toward Reliable Density Functional Methods without Adjustable Parameters: The PBE0 Model. *J. Chem. Phys.* **1999**, *110*, 6158–6170.

(5) Perdew, J. P.; Ernzerhof, M.; Burke, K. Rationale for Mixing Exact Exchange with Density Functional Approximations. *J. Chem. Phys.* **1996**, *105*, 9982–9985.

(6) Parrish, R. M. et al. Psi4 1.1: An Open-Source Electronic Structure Program Emphasizing Automation, Advanced Libraries, and Interoperability. *J. Chem. Theory Comput.* **2017**, *13*, 3185–3197.

(7) Smith, D. G. et al. PSI4 1.4: Open-Source Software for High-Throughput Quantum Chemistry. *J. Chem. Phys.* **2020**, *152*, 184108.

(8) Turney, J. M. et al. Psi4: An Open-Source Ab Initio Electronic Structure Program. *Wiley Interdiscip. Rev.: Comput. Mol. Sci.* **2012**, *2*, 556–565.

(9) Dunning, T. H. Gaussian Basis Sets for Use in Correlated Molecular Calculations. I. The Atoms Boron Through Neon and Hydrogen. *J. Chem. Phys.* **1989**, *90*, 1007–1023.

(10) Kendall, R. A.; Dunning, T. H.; Harrison, R. J. Electron Affinities of the First-row Atoms Revisited. Systematic Basis Sets and Wave Functions. *J. Chem. Phys.* **1992**, *96*, 6796–6806.

(11) Woon, D. E.; Dunning, T. H. Gaussian Basis Sets for Use in Correlated Molecular Calculations. IV. Calculation of Static Electrical Response Properties. *J. Chem. Phys.* **1994**, *100*, 2975–2988.

(12) Weigend, F. A Fully Direct RI-HF Algorithm: Implementation, Optimised Auxiliary Basis Sets, Demonstration of Accuracy and Efficiency. *Phys. Chem. Chem. Phys.* **2002**, *4*, 4285–4291.

(13) Weigend, F.; Köhn, A.; Hättig, C. Efficient Use of the Correlation Consistent Basis Sets in Resolution of the Identity MP2 Calculations. *J. Chem. Phys.* **2002**, *116*, 3175–3183.

(14) Mulvey, D. M.; Jordan, K. D.; Rutter, M. J.; Misquitta, A. J. Unpublished Results. **2024**.

(15) Harris, C. R. et al. Array Programming with NumPy. *Nature* **2020**, *585*, 357–362.
